# Tobacco in Hysteria

**Published:** 1842-06

**Authors:** J. H. Thompson

**Affiliations:** Salem, N. J.


*Tobacco in Hysteria.* By Dr. J. H. Thompson, of Salem, N. J.—August 19, 1839.—Frances S., æt. 22, unmarried, dark complexion, black hair, moderate embonpoint, about the ordinary height, after having performed an almost incredible day's work, while her clothes were literally "soaked" with perspiration, sat down, with her bare feet resting upon a cold pavement, and continued in this situation for half an hour or more, until she began to "feel strangely," and as if something was "rising in her throat." She walked into the house and was immediately attacked with violent hysterical convulsions. I saw her at eight o'clock, P. M., about an hour after the attack. She was then upon a bed, surrounded by a number of persons, who, as usual, appeared to think the convulsions could be arrested by *main force*, since their utmost efforts had been directed to the injudicious and unsuccessful attempt of preventing any motion on the part of the patient. Her arms were thrown violently in every direction; her head was forced backward, and with the body formed a complete arch. The musclcs of the neck and trunk were under rigid tonic contraction, while those of the lower extremities were not at all affected. The face was swelled; the eyes firmly closed; jaws could be opened with difficulty, but were quickly closed with a loud "snap." Some abortive attempts were made to introduce medicine into her stomach; her feet were placed in stimulating pediluvia, and cold applications were made to the head which was extremely hot. No impression had been produced upon the disease. I tied up her arms and made a free opening into the vein, from which the blood flowed in a large stream. No regard was paid to mere quantity, its effects alone were considered. In a short time the convulsive motions ceased; the face began to lose the dark flush which had overspread it; the muscular system gradually relaxed, and the first intelligible words spoken by the patient since the attack were, "I feel sick." About forty ounces of blood had been abstracted.

20. Patient slept a little last night. Feels this morning, to use her own expression, "as if every bone had been broken and every joint dislocated." Says the catamenia appeared a week ago, and were as usual. Bowels were operated upon by enamenta. Still feels the globus hystericus occasionally in a slight degree. Antispasmodics freely administered during the day. At about the same hour as on the preceding day the patient was again attacked. Assafœtida, ʒ j. with 60 gtts. of tinct. opii, was given in enema, and repeated two or three times without effect. Pulse strong; vein again opened; with the loss of 20 ounces of blood, the convulsions ceased.

These attacks occurred at the same hour the three following days, notwithstanding the liberal use of almost the whole list of antispasmodics, and other remedies, amongst which quinine was given, as the disease appeared to have assumed a periodical character. The convulsions appeared to increase in violence: they lasted for several hours, and left the patient in an extremely exhausted condition. During the attack, her countenance was so altered in appearance and expression that her most intimate friends could not have recognized her. Her throat was the seat of chief distress; desperate and continual efforts were made, as if to tear away something that was chocking her. A distressing "clucking" noise was made, as if the glottis was spasmodically opened and closed. Under these circumstauces I determined to make a trial of the powers of tobacco. On the next attack some leaves were procured. One was placed for a few minutes in hot water, and then spread over the epigastric region of the patient. In fifteen minutes the hysterical symptoms had all disappeared. The patient felt sick and continued so for some time, but did not vomit. At the usual hour on the following day, and also on the day after, she was again seized, but on both occasions the attack was arrested *in limine*, by the tobacco, and returned no more. No other means were employed. The patient slowly returned to her former state of health.

This is but a solitary instance of the use of tobacco in one of the Protean forms of this disease, and I am by no means disposed to place much reliance upon isolated cases. The facts are given as they occurred. It will be for future experience to confirm the efficacy of the remedy, or to reject it as unworthy of confidence in this disease.—*Am. Jour. of Med. Sciences.*

